# Size-Mediated Interaction between a Cushion Species and Other Non-cushion Species at High Elevations of the Hengduan Mountains, SW China

**DOI:** 10.3389/fpls.2017.00465

**Published:** 2017-04-05

**Authors:** Yang Yang, Jian-Guo Chen, Christian Schöb, Hang Sun

**Affiliations:** ^1^Key Laboratory for Plant Diversity and Biogeography of East Asia, Kunming Institute of Botany, Chinese Academy of SciencesKunming, China; ^2^Department of Evolutionary Biology and Environmental Studies, University of ZürichZürich, Switzerland

**Keywords:** plant–plant interaction, facilitation, plant–soil feedback, soil quality, cushion plant, Himalaya-Hengduan Mountains

## Abstract

*Arenaria polytrichoides* (Caryophyllaceae) is a common cushion plant occurring at high elevations in the Himalaya-Hengduan Mountains, SW China. It frequently has other non-cushion species growing within its canopy, forming a contrast with the surrounding areas because it creates patches of higher diversity and greater biomass. In this study, we examined the relationship between the cushions and associated non-cushion species along a gradient of cushion size. A total of 200 *A. polytrichoides* individuals were selected to fit four size classes. Field measurements were carried out to assess canopy structure, functional traits relevant to growth and reproduction, and soil quality below cushions along the size gradient. Furthermore, the size effect of cushions on the richness and abundance of species and biomass production was also examined. All the morphological variables examined exhibited a positive correlation with cushion size, as did the nutrients under cushions. Large and compact cushions were associated with higher soil nutrient contents compared with small and loose cushions. As a result of these biogenic environmental changes, there was a stronger facilitation effect performed by large cushions. Data pertaining to functional traits revealed that large cushions benefit from the enhanced resources within their compact structure and exhibit greater fitness and a higher reproductive output than small cushions. Our data indicated that interactions occur between cushion species and other plants depending on the size of the cushions, probably because of the greater heterogeneity of conditions beneath larger cushions. These findings provide a clear demonstration of the generally overlooked importance of the traits of nurse plants, such as size and age, in terms of their facilitative effects.

## Introduction

Facilitative effects can strongly influence the composition, diversity, and functions of natural plant communities in ecosystems such as desert, Mediterranean, alpine, and coastal habitats (Callaway, 2007). These positive plant–plant interactions depend on the specific combinations of nurse and beneficiary species and the underlying environmental conditions (Pistón et al., 2016). It is widely accepted that facilitation and competition operate simultaneously. According to the stress gradient hypothesis (SGH), competition should be relatively more frequent in low stress conditions and facilitation more frequent in high stress conditions (Bertness and Callaway, 1994). This hypothesis has been tested in various studies examining climatic gradients on comparatively large spatial or geographical scales and at finer local spatial scales along resource gradients (e.g., Choler et al., 2001; Callaway et al., 2002; Cavieres and Badano, 2009). In all of these studies, the shift from competition to facilitation was related to changes in the ambient environmental conditions surrounding both benefactor and beneficiary species (but see Maestre et al., 2009). Recently, some studies have suggested that the interplay between facilitation and competition can shrift during the ontogenetic life cycle of both nurse and beneficiary species (e.g., Pugnaire et al., 1996; Miriti, 2006; Reisman-Berman, 2007; Soliveres et al., 2010; Allegrezza et al., 2016). For instance, in a semi-arid habitat in Spain, Pugnaire et al. (1996) reported that plant diversity in the understory beneath a leafless leguminous shrub, *Retama sphaerocarpa*, increased with the shrub’s age and size. They considered that this was probably due to the greater heterogeneity under larger canopies. In the shrubland of the transition area between the Mediterranean and the semi-arid zone of Israel, Reisman-Berman (2007) found that facilitation by the spiny shrub *Sarcopoterium spinosum* resulted in a unimodal pattern of conspecific recruitment in relation to canopy density and age. In addition, many studies have focused on the variable effects of beneficiary species on nurse plants. For example, a common shrub in the Colorado Desert of California, USA, *Ambrosia dumosa*, exhibits an ontogenetic shift in response to adult neighbors: there is facilitation of seedlings and juveniles by adult neighbors, while there is competition between larger plants and their adult neighbors (Miriti, 2006). These studies highlight the importance of a more critical examination of ontogenetic shifts and the need to take these into account when considering plant–plant interactions. Ignoring ontogenetic shifts in interactions could lead to incorrect conclusions being drawn about the various outcomes of plant–plant interactions on environmental gradients (Callaway, 2007).

In alpine regions, pioneer cushion species commonly act as typical nurse plants through ameliorating microclimatic conditions within their compact structure (Reid et al., 2010) and the positive effect of soil biota (e.g., microbial endophytes) inhabiting roots and/or rhizosphrere of cushions (Molina-Montenegro et al., 2015). Cushion species can provide a thermal buffer, hydric refuge, islands of fertility and microorganism associations for establishment and recruitment of other non-cushion species (e.g., Cavieres et al., 2006; Yang et al., 2010; Casanova-Katny et al., 2011; Molina-Montenegro et al., 2015). Thus, by increasing species richness, cushion species play an important role in enhancing the diversity of harsh alpine environments at community level both globally and regionally (Cavieres and Badano, 2009; Cavieres et al., 2014; Chen et al., 2015). According with the predictions of the SGH, increasing frequency, intensity and importance of positive interactions with increasing level of abiotic stress have been reported in most studies of facilitation by cushion species (e.g., Cavieres et al., 2006; Cavieres and Badano, 2009; Yang et al., 2010). However, the facilitative effects of cushion species on beneficiary species can change with development and maturation of the beneficiary (le Roux et al., 2013). Unlike the majority of studies showing a size-dependent and ontogenetic shift from facilitation during juvenile establishment to interference or a neutral effect on adult plants, le Roux et al. (2013) reported that the facilitative effects of *Azorella selago* cushions on *Agrostis magellanica* were strongest for middle-sized individuals of *A. magellanica*.

The facilitative effects of cushions on other plant species are linked to the modifications of environments beneath them. Therefore, changes in canopy traits may affect the facilitative effect of the cushion species (Schöb et al., 2013). In the Sierra Nevada Mountains of Spain, Schöb et al. (2013) found that changes in the physiology of *Arenaria tetraquetra* cushions in response to variations in abiotic factors along a stress gradient can affect the species’ morphology (i.e., size and compactness), which in turn influences the microenvironments created by cushions and, eventually, modulates the outcome of their interaction with other non-cushion species. As an adaptation to harsh and unpredictable environmental conditions, alpine cushion species often appear to have long life spans, during which their canopy structure and/or physiological status changes in response to ontogenetic processes. Based on the size–age relationship, some studies have used plant size represented by canopy diameter to estimate a cushion’s longevity (Morris and Doak, 1998; Kleier and Rundel, 2004; le Roux and McGeoch, 2004). Within a single mixed-size population of cushion species in a given environment, one might expect to find a size-related interaction between cushion species and non-cushion species as a result of changed functional traits of the cushions and their biogenic effects on microclimate. However, to date, this hypothesis has not been tested.

In this study, we test the hypothesis that size-related changes in canopy structure and subsequent physiological status can alter the facilitative effects of *Arenaria polytrichoides* on co-occurring plant species at high elevations in the Himalaya-Hengduan Mountains (HHM). HMM has been identifies as a hotspot for temperate biodiversity (Mittermeier et al., 1999), supporting the globally richest alpine flora (Xu et al., 2014) where facilitation by cushion plants is key to structure diversity and composition of natural communities in these species-rich alpine habitats (Chen et al., 2015). It has been suggested that for potential nurse plants to facilitate other plants effectively, specific morphological traits and an adequate size are required to provide shelter and/or protect from hostile conditions (Incerti et al., 2013). One previous study indicated the importance of facilitative effect of *A. polytrichoides* cushions on increasing species richness in the alpine zone of the HHM and attributed this to nutrient enrichment under cushions at high elevation sites (Yang et al., 2010). For enhanced soil fertility under the nurse canopy of cushion species there has to have been a sufficiently long period for nutrients to accumulate, implying life spans of decades or even centuries. We therefore argue that a small and not well-developed *A. polytrichoides* cushion is likely to provide negligible nurse effects on other plant species. It has been shown that facilitation by nurse plants can be strongly influenced by plant size, with larger individuals exerting a stronger positive impact on modification of surrounding environments and consequently on the growth of their neighbors (Allegrezza et al., 2016). With canopy development, fertility beneath the cushion should increase such that its facilitative effects should also increase. We thus predict that density and growth of new recruits of beneficiary species will vary according to the size-structure of the studied cushion population, with high densities and larger individuals of new recruits occurring in association with large and well-developed cushions.

## Materials and Methods

### Study Area

The study site (N28°22′, E99°58′) is located at an elevation of 4900 m at the Baima pass on the Baima Snow Mountains, Deqen County, Yunnan Province, SW China. This site was chosen because it supports a population of cushion species of a variety of sizes, and also because facilitation by *A. polytrichoides* cushions has already been shown to be frequent and important in natural alpine plant communities of this region (Yang et al., 2010; Chen et al., 2014, 2015).

At high elevations in the HHM, the summer climate is monsoonal and characterized by cold rain or sleet, interrupted by short periods of intense solar radiation (Yang et al., 2008). Annual precipitation recorded from 1982 to 1984 at the nearest meteorological station (N28°23′, E99°01′, 4290 m elevation) was 680–790 mm, with over 600 mm falling during the growing season from the early to middle of June until the end of September. The annual average precipitation in the study region increases with altitude at 36.3 mm per 100 m. The annual average air temperature is -1.0°C, with 6–8°C during growth season (Wang, 2006).

Air temperature (°C) and relative humidity (RH, %) at 15 cm above the soil surface at the study site recorded every 30 min by using an integrated thermistor (Hobo, Pro V2, Onset Computer Crop, Cape Cod, MA, USA) indicated average temperature during the growing season from middle of June to the end of September 2015 was 4.87 ± 0.04°C (mean ± standard error [s.e.], and thereafter), and average RH during daytime (0800–2000 h) of that period was 89.9 ± 0.24%. Absolute maximum and minimum temperatures were 17.80°C and -1.95°C recorded in the middle (June, 24) and end (September, 29) of the growing season 2015, respectively.

### Study Species

*Arenaria polytrichoides* (Caryophyllaceae) is a long-lived and stress tolerant perennial herb that forms a hemispherical cushion; it is mainly found at high elevations in the HHM. Like most species with a cushion life form, *A. polytrichoides* cushions retain their dead leaves under the compact structure and these can be seen as litter and a humus trap. The dense canopy structure of *A. polytrichoides* cushions has been shown to result in soil below with a higher moisture and nutrient content than adjacent open areas and the cushions provide a more favorable microenvironment for many other non-cushion plants (Yang et al., 2010; Chen et al., 2014). At the study site, the relative cover of *A. polytrichoides* was determined along five 10 m linear transects with a distance between transects of 5 m; it ranged between ca. 8 and 14%.

### Sampling of Species and Biomass in Cushions and Cushion-Free Areas

Cushion plants normally have a roughly elliptical shape. To obtain comparable sizes for examining species richness, abundance and biomass production in patches sampled in cushions and paired surrounding open areas (i.e., areas not covered by cushion, see, e.g., Cavieres et al., 2014), metallic hoops with diameters of 10, 15, 20, and 30 cm (i.e., class I, II, III, IV) were prepared previously. At each selected cushion, a metallic hoop of similar diameter was placed on the surface of cushions and individual plants found inside the hoop were counted. In addition, aerial parts of species within the hoop of each cushion patch were collected and stored in individual paper bags (i.e., one bag per species per sample). Then, the same metallic hoop was placed at random on the open areas at least 1 m away from the cushion, where again we recorded and collected all plants. Fifty cushions were selected for each of the four size classes. Accordingly, 200 randomly distributed *A. polytrichoides* cushions were selected within ∼1 ha of study site. Bags containing the biomass samples were placed in a drying oven at 75°C for 72 h before being weighed to determine the aboveground dry biomass (mg) of each species within each sample. All the diversity and biomass sampling was undertaken in the middle of August 2015 at the peak of the growing season.

In addition, the height and the maximum (a, cm) and minimum (b, cm) canopy diameter of each cushion were measured. The canopy area (cm^2^) and volume (cm^3^) of each cushion were calculated using following equations:

Area=(π×a×b)/4,

where *a* and *b* are mean maximum and minimum canopy diameter (cm), respectively.

Volume=4/3×π×a×b×c,

where *a*, *b*, and *c* are mean maximum and minimum canopy diameter (cm) and height of canopy (cm), respectively.

To compare species richness of sampled patches of cushions and paired open areas in each size class, we generated a species × samples matrix containing each sampled cushion and open area of each size class, where each cell (*i, j*) contained the abundance of the *i*th species in the *j*th sample. We then used this to determine the effect of cushion plants on species richness within and outside cushions in each of the four size classes. From the matrices, 500 samples were randomly drawn, with replacement, for each sample size (from one sample to the maximum number of samples); then the species richness of the 500 samples was calculated using Coleman’s algorithm (Coleman et al., 1982). To avoid bias due to differences in the samples that were replaced, we ran the rarefaction analysis for cushion and open areas at each size class 20 times. Maximum likelihood estimates of the species richness of cushions and open areas at the asymptote of the 20 sample-based rarefaction curves were averaged and plotted. The rarefaction analyses were carried out with EstimateS v. 9.01 software (Colwell, 2000, University of Connecticut, Storrs, CT, USA). The increase in species richness for each size class due to the presence of *Arenaria* cushions (ISR) was calculated as:

$$ISR=[{(S}_{c}-S_{0})/S_{0}]\times 100\%$$

where *S*_c_ and *S*_o_ are estimated values for species richness in cushion and open areas plots, respectively, at the asymptotes of sample-based rarefaction curves (Cavieres et al., 2014). The estimated species richness between cushions and its corresponding open plot across the four size classes were tested with one-way ANOVA and *post hoc* multiple comparisons (LSD).

In order to examine the size effect of cushion on the abundance and biomass production of non-cushion species, we calculated the relative interaction index (RII_abundance_ and RII_biomass_) for each non-cushion species recorded in association with the canopy of cushions in the four size classes, separately. For each cushion size class, RII_abundance_ and RII_biomass_ were calculated as follows:

$${RII}_{abundance}\,\;or\,{RII}_{biomass}=\frac{\left(\#\,\;with\,\;\,cushion\,\;species-\#\,\;in\,\;open\,\;areas\,\right)}{\left(\#\,\;with\,\;\,cushion\,\;species+\#\,\;in\,\;open\,\;areas\,\right)}\,$$

where # indicates the number of individuals/biomass of each non-cushion species for RII_abundance_ or RII_biomass_, respectively. Thus, RII_abundance_ or RII_biomass_ = 1, when all individuals of a species occur within cushions; RII_abundance_ or RII_biomass_ = -1, when all individuals of a species occur within cushion-free areas; RII_abundance_ or RII_biomass_ = 0, when all individuals of a species are distributed equally/produced same amount of biomass between cushions and open areas. Mean values of RII_abundance_ or RII_biomass_ across all species within the four cushion size classes were then used as an estimate for the average size effect of the studied *Arenaria* cushions on abundance and biomass production of other plant species at the study site. One sample *t*-test was used to examine if RII_abundance_ or RII_biomass_ values of cushions at each of four size classes were different from zero.

### Micro-Environmental Data

Between 14 and 15 August 2015, 10 samples of topsoil (0–15 cm) beneath cushions of each size class and in open areas were collected. These soil samples were stored in sealed containers and fresh mass was determined immediately after sampling before being transported to the laboratory within 24 h. Gravimetric soil water content (SWC, %) of five samples of cushions of each size class and open areas were measured by mass loss after drying at 105°C for 72 h. Bulk density (g cm^-3^) was determined by weighing a densely packed volume of these dry soils. The remaining five soil samples from underneath cushions of each size class and in the open areas were sent to the Laboratory for Soil Analysis at the Agricultural Institute of Yunnan, where soil organic matter (SOM, g kg^-1^), available nitrogen (NH_4_^+^ and NO_3_^-^, mg kg^-1^), phosphorus (mg kg^-1^), and potassium (mg kg^-1^) were measured by using potassium dichromate heating oxidation-volumetric method (for SOM), alkali diffusion method (for available nitrogen), sodium bicarbonate extraction Mo-Sb Antispetrophotography method (for available phosphorus), neutral normal salt ammonium acetate extraction flame photometer method, and boiling nitric acid method (for available potassium), respectively. Because litter is an important source of N for cushion plants at high elevations (He et al., 2014), litter depth for five cushions at each size class was also examined by penetrating the selected cushion on its vertical axis with a metallic rod and measuring the distance (cm) between the litter layer under the outer leaves and the soil underneath. The differences between soil characteristics (i.e., SWC, SOM, availability of N, P, K) and the presence/absence of cushions and cushion size were examined statistically using one-way ANOVA and *post hoc* multiple comparisons (LSD/Tamhane). In addition, the differences between litter depths of cushions of different sizes were also examined with one-way ANOVA and *post hoc* multiple comparisons (LSD).

### Plant Functional Traits

Variations in four functional traits relevant to growth, physiology and reproduction that could respond to ontogenetic processes were assessed for cushions in each of the four size classes. The traits measured were:

(1) Leaf mass per area (LMA) (g m^-2^), i.e., the ratio between leaf dry mass and fresh leaf area. LMA exhibits inherent variations between different functional groups (e.g., ferns, herbs, graminoids, shrubs, trees) and responds to environmental conditions (e.g., radiation, atmospheric composition, nutrient), leaf position and leaf/plant age (reviewed by Poorter et al., 2009). LMA is a key trait in plant growth and an important indicator of plant strategies. LMA is positively correlated with lifespan of leaves and roots (Ryser, 1996; Wright et al., 2004). Species with an inherently high LMA are more efficient at conserving acquired nutrients and carbon and thereby have a fitness advantage under adverse growing conditions (Poorter et al., 2009). Within a given species, there is often a strong positive relationship between photosynthetic capacity and LMA (Poorter et al., 2009). Anatomically, species with high LMA have a thicker leaf blade and/or denser tissues. In addition, leaf chemical content (e.g., protein, lignin, lipid, total non-structural carbohydrates) is positively related to LMA (Poorter et al., 2009). (2) Leaf dry matter content, LDMC (g kg^-1^), i.e., the ratio between leaf dry mass and fully rehydrated fresh mass. LDMC is generally positively correlated with life span and negatively correlated with potential relative growth rate (RGR) and likelihood of physical damage. LMA and LDMC were measured in leaves of five cushions of each size class. (3) Foliar δ13C (‰) was determined using an isotope ratio mass spectrometer (Isoprime 100, UK). Leaf δ13C values are commonly used for assessing plant photosynthetic activity and water use efficiency. Variations in leaf δ13C are caused by environmental factors (e.g., soil moisture, irradiance, atmospheric CO_2_ concentrations) and morphology (e.g., leaf size, thickness, canopy height). These morphological factors explain most variations in leaf δ13C with respect to plant phenology, development and age (reviewed by Dawson et al., 2002). Foliar δ13C was measured for five cushions of each size class. (4) The number of flowers per area (FNA), counted in three randomly placed 2 cm × 2 cm quadrats on each cushion of each size class and used as a proxy for reproductive effort. FNA was recorded for 20 cushions of each size class between 10 and 15 June 2015, when most individuals of *A. polytrichoides* were in flowering. The differences between functional traits relevant to physiology, vegetative and reproductive growth of *Arenaria* cushions of different size classes were examined statistically using one-way ANOVA and *post hoc* multiple comparisons (LSD/Tamhane).

There are several architectural traits that can vary in response to ontogenetic processes and could act as mechanisms for variation in establishment and recruitment success of other plant species as a result of their effects on the micro-environment within the canopy of cushions (Crutsinger et al., 2010; Michalet et al., 2011). In this study, four functional traits were measured and used as proxies of cushion size and biomass, as suggested by Schöb et al. (2013): (1) canopy diameter (cm) was used as proxy for plant size; (2) cushion thickness was measured by inserting a metal rod into the selected cushion and measuring the mean distance (cm) between the tip of the outer leaves and the soil underneath the plant at five randomly placed positions within the cushion; (3) branch density (branches cm^-2^) was calculated from the number of terminal branches counted in one randomly placed 2 cm × 2 cm quadrat per cushion; (4) leaf density (leaves cm^-2^) was calculated as the average number of leaves per terminal branch of 10 randomly selected branches multiplied by the number of branches cm^-2^. High values of branch and leaf density indicated high cushion compactness. In addition, these functional traits were considered to reflect the physiological status of the plants themselves (Milla et al., 2008). All these traits were measured on the same individuals for six cushions of each class size between 16 and 19 August 2015.

For the four functional traits used as proxies for plant morphology, a principle component analysis (PCA) with Varimax rotation was used to reduce the number and multicollinearity of variables (Schöb et al., 2013). The principal components (PCs) were then employed as predictor variables in a linear model, testing the relationship between cushion morphology and differences in soil properties (SWC, bulk density, SOM, availability of N, P, and K) beneath cushions and in open areas and plant traits (LMA, LDMC, flower number per area) with growth, physiological and reproductive relevance. Because soil properties and physiological and reproductive traits of cushions were not from the same individuals, we calculated means for each of these variables per size class to test for their relationships. These observed relationships were used as an indication of the role of cushion morphology in the potential effects of cushions on micro-environmental conditions. Using a similar model, we also examined the relationship of RII_abundance_ and RII_biomass_ with cushion morphology.

## Results

### Diversity and Biomass Production

**FIGURE 1:**
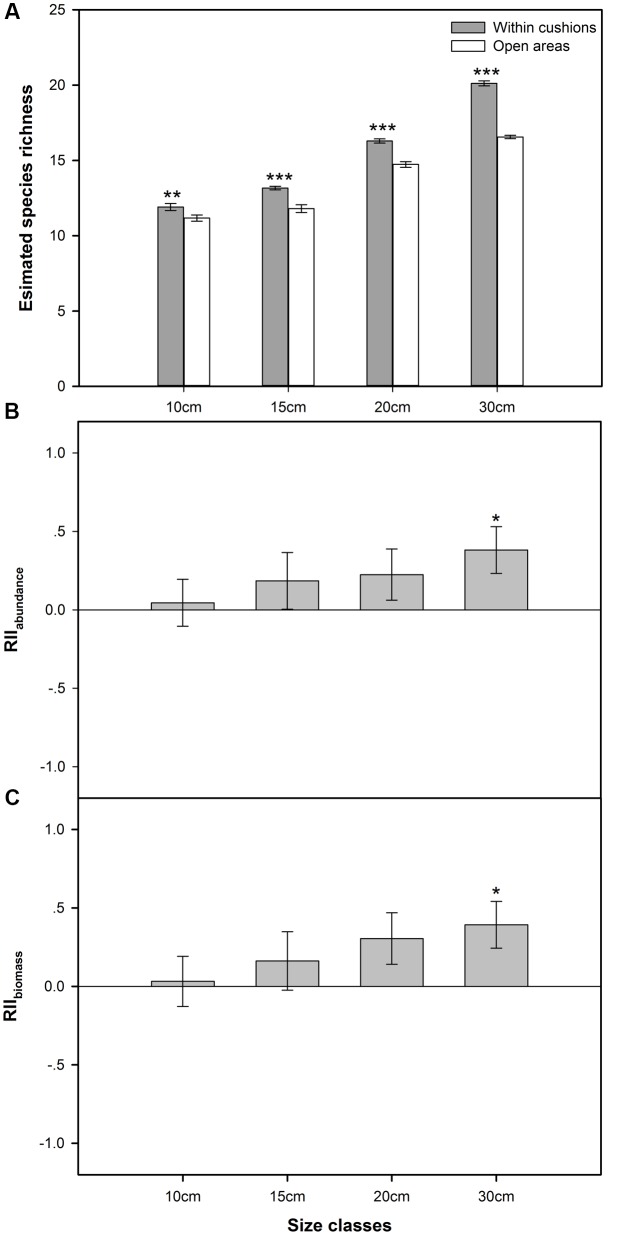
**(A)** The number of species estimated by rarefaction curves (mean ± SE) within cushions and open areas at each size class (*n* = 20, the number of replicated rarefaction analyses). **(B)** Mean relative index of RII_abundance_ of cushions at each of four size classes (mean ± SE, *n* = the number of non-cushion species); **(C)** Mean relative index of RII_biomass_ of cushions at each of four size classes (mean ± SE, *n* = the number of non-cushion species). ^∗^*p* < 0.05, ^∗∗^*p* < 0.01, ^∗∗∗^*p* < 0.001.

There was significantly higher species richness recorded in sampled cushions than open areas for all the four size classes (*F* = 240.855, *p* < 0.001, also see **Figure 1A**). ISR increased with cushion size, ranging from ca. 6.5% with smallest cushions to 21.5% with largest cushions (for cushions in size class II and III, these values were 11.6 and 10.5%, respectively).

Both mean values of RII_abundance_ and RII_biomass_ were positive for cushions in all four size classes (**Figures 1B,C**). Overall, both RII_abundance_ and RII_biomass_ were positively related to cushion size (**Figures 1B,C**), with the lowest mean values of both RII_abundance_ (0.04) and RII_biomass_ (0.03) associated with the smallest cushions (class I), and the highest values of RII_abundance_ (0.38) and RII_biomass_ (0.39) with the largest cushions in class IV (**Figures 1B,C**). For the smallest cushion size class, 10 and 11 of 17 species (ca. 58 and 65% of all the species) recorded exhibited positive RII_abundance_ and RII_biomass_ values (Supplementary Table 1). Thus, even the smallest cushions seem to have a positive impact on the abundance and biomass of the majority of species within the community. These values increased to 68% (17 of 25 species) and 72% (18 of 25 species) in the largest cushions (Supplementary Table 1).

Generally, a decreasing *P*-value in the one sample *t*-tests for both RII_abundance_ and RII_biomass_ was observed from size class I to IV. For RII_abundance_, significant differences from zero were found for cushions in class IV (*p* = 0.017), but not for cushions in class I (*p* = 0.768), II (*p* = 0.324), and III (*p* = 0.183). For RII_biomass_, significant/marginally significant difference was found for cushions in the size classes III (*p* = 0.078) and IV (*p* = 0.017), but not in classes I (*p* = 0.768) and II (*p* = 0.324).

### Effects of Arenaria Cushions on Soil Quality

With the exception of nitrogen availability, for which *p* = 0.584, values for SWC, bulk density, SOM, and the availability of P and K in soil under the smallest cushions in class I were significantly/marginally significantly higher than in open areas (**Table 1**). In contrast, all examined soil properties under the cushions of classes II, III, and IV had (marginally) significantly higher values than they did under the smallest cushions (**Table 1**). Cushions in class IV had marginally significantly higher soil potassium availability under them than soil under the cushions in class III (*p* = 0.091, **Table 1**). A significant difference in litter depth between open areas and cushions in different size classes was found (*F* = 77.216, *p* < 0.001). Pairwise tests indicated greater depth beneath large cushions than small cushions (**Table 1**).

**Table 1:** Properties of the topsoil (0–15 cm) under the canopy of *Arenaria polytrichoides* cushions in four size classes and in the corresponding open area outside the canopy.

		Class			
Variable	Open areas	I	II	III	IV
SWC (%)	11.95 ± 0.81^a^	19.42 ± 2.84^b^	16.46 ± 1.70^ab^	28.11 ± 2.92^c^	23.78 ± 2.99^bc^
SOM (g Kg^-1^)	62.11 ± 6.05^a^	101.13 ± 12.59^b^	95.19 ± 8.67^b^	143.60 ± 17.48^c^	132.75 ± 11.74^c^
Availability of nitrogen (mg Kg^-1^)	193.63 ± 16.46^a^	212.82 ± 34.25^a^	264.96 ± 24.16^b^	378.06 ± 25.27^c^	331.89 ± 17.64^c^
Availability of phosphorus (mg Kg^-1^)	6.02 ± 0.67^a^	9.31 ± 1.21^b^	10.01 ± 0.87^b^	13.49 ± 0.82^c^	15.68 ± 1.65^c^
Availability of potassium (mg Kg^-1^)	15.80 ± 1.24^a^	36.60 ± 3.76^b^	32.20 ± 4.68^b^	39.20 ± 2.69^bc^	47.00 ± 1.87^c^
Litter depth (cm)	0.00 ± 0.00^a^	3.95 ± 0.12^b^	6.57 ± 0.20^c^	7.99 ± 0.32^d^	9.12 ± 0.69^e^
Bulk density (g cm^-3^)	0.99 ± 0.04^a^	0.76 ± 0.06^b^	0.82 ± 0.02^b^	0.58 ± 0.05^c^	0.62 ± 0.06^c^

*For each parameter, values are mean ± standard error (*n* = 5). Different letters indicate significantly different means. SWC, gravimetric soil water content; SOM, soil organic matter*.

### Cushion Morphology and Physiological Traits

The selected cushions in the four size classes differed significantly in lateral spread, thickness, canopy area and volume (**Table 2**). Other examined morphological traits (i.e., leaf density and branch density) also increased with cushion size (**Table 2**).

**Table 2:** Morphological traits of *Arenaria polytrichoides* cushions grouped into four size classes.

	Class			
Variable	I	II	III	IV
Canopy area (cm^2^)	0.91 ± 0.02^a^	1.81 ± 0.03^b^	3.86 ± 0.07^c^	6.61 ± 0.12^d^
Canopy volume (cm^3^)	1.86 ± 0.08^a^	5.75 ± 0.18^b^	14.56 ± 0.49^c^	31.74 ± 1.15^d^
Canopy dry mass (g)	32.45 ± 3.84^a^	244.66 ± 43.06^b^	443.06 ± 65.75^c^	509.89 ± 42.27^c^
Lateral spread (cm)	10.00 ± 0.32^a^	18.00 ± 0.83^a^	22.00 ± 0.89^b^	29.40 ± 0.25^b^
Thickness (cm)	3.46 ± 0.20^a^	6.26 ± 0.22^b^	7.67 ± 0.44^c^	8.62 ± 0.33^d^
Branch density (branches cm^-2^)	6.50 ± 0.40^a^	7.50 ± 0.35^a^	7.70 ± 0.46^b^	9.85 ± 0.45^c^
Leaf density (leaves cm^-2^)	55.04 ± 4.67^a^	60.10 ± 3.84^a^	62.25 ± 3.30^a^	79.62 ± 4.81^b^

*For each parameter, values are mean ± standard error (*n* = 50 for canopy area and canopy volume, while *n* = 6 for lateral spread, thickness, leaf density, and branch density). All examined morphological traits were significantly different between cushions in different size classes (Canopy area: *F* = 1681.217, *p* < 0.001; Canopy volume: *F* = 1008.474, *p* < 0.001; Canopy dry mass: 19.285, *p* < 0.001; Lateral spread: 157.647, *p* < 0.001; Thickness: *F* = 50.118, *p* < 0.001, Branch density: *F* = 11.356, *p* < 0.001; Leaf density: *F* = 6.884, *p* < 0.01). For each size class, superscripts with different letters indicate significantly different means*.

Although no significant difference in LMA was found between the four size classes (*F* = 1.770, *p* = 0.199), the *post hoc* test indicated a significantly higher LMA value for cushions in class IV (*p* = 0.042) than cushions in class I. Overall, a significant size effect on LDMC (*F* = 5.383, *p* = 0.011) was observed across the four size classes of *Arenaria* cushions, with increased LDMC with increasing cushion size (**Table 3**). In addition, pairwise testing revealed significantly lower LDMC values for cushions in class I than cushions in class II (*p* = 0.006), class III (*p* = 0.033), and class IV (*p* = 0.002). No significant difference in foliar δ13C (*F* = 1.267, *p* = 0.319) was found across the four size classes. A significant difference in flower number per area (FNA) was found between the cushion size classes (*F* = 3.085, *p* = 0.032, **Table 3**), with a significantly higher FNA value for cushions in class IV than those in class I (*p* = 0.039) and class II (*p* = 0.007). The FNA value for cushions in class III was marginally significantly higher than for cushions in class II (*p* = 0.059).

**Table 3:** Physiological and reproductive traits of *Arenaria polytrichoides* cushions grouped into four size classes.

	Class			
Variable	I	II	III	IV
LMA (g m^-2^)	33.93 ± 1.95^a^	36.32 ± 1.57^ab^	37.28 ± 1.45^ab^	38.51 ± 0.78^b^
LDMC (g kg^-1^)	161.69 ± 4.24^a^	202.59 ± 13.96^b^	188.30 ± 5.83^b^	205.77 ± 9.08^b^
The number of flowers per area (flower cm^-2^)	5.62 ± 0.38^a^	5.16 ± 0.22^a^	6.42 ± 0.59^a^	6.63 ± 0.29^b^
Foliar δ13C (‰)	-26.71 ± 0.29^a^	-26.10 ± 0.31^a^	-26.17 ± 0.22^a^	-26.42 ± 0.08^a^

*For each parameter, values are mean ± standard error (*n* = 5). For each size class, superscripts with different letters indicate significantly different means. LMA, leaf mass per area; LDMC, leaf dry matter content*.

### Cushion Morphology and Diversity/Biomass

Overall, all functional traits used as proxies for cushion morphology varied with size class (**Table 2**). Bigger cushions were larger, thicker and had higher branch and leaf density than smaller cushions (**Table 2**). The first three axes of the PCA based on the four morphological traits explained ca. 46.4, 28.9, and 23.2% of the variance, respectively. The first axis (PC1) represents cushion size (PC1_lateralspread_ = 0.859) and thickness (PC1_thickness_ = 0.924). Both the second (PC2) and third (PC3) axes represent cushion compactness (PC2_leafdensity_ = 0.867, PC3_branchnumber_ = 0.787).

Both RII_abundance_ (*r*^2^ = 0.800, *p* = 0.100, *n* = 4 [i.e., the four size classes] here and thereafter) and RII_biomass_ (*r*^2^ = 0.919 *p* = 0.042) showed a significant/marginally significant positive relationship with PC1. ISR was significantly and marginally significantly positively related with PC3 (*r*^2^ = 0.942, *p* = 0.029) and a model including PC2 and PC3 (*r*^2^ = 0.866, *p* = 0.069), respectively. The absolute difference in available N between cushions of different size classes and open areas exhibited a marginally significant positive correlation with PC1 (*r*^2^ = 0.852, *p* = 0.077). PC1 was also the best predictor of the changes in LMA (*r*^2^ = 0.947, *p* = 0.027). The model including PC2 and PC3 was marginally significantly related with the absolute difference in available K (*r*^2^ = 0.854, *p* = 0.076) between cushions of different size classes and open areas.

## Discussion

Previous studies indicated that the facilitative role of cushion plants at high elevations of the HHM relies on their ability to modify the surrounding environments, particularly soil nutrients (Yang et al., 2010). Here, we showed that the ‘islands’ of high fertility created by cushion species are heterogeneous, producing a soil quality gradient shaped by variations in physiological status and canopy structure. Moreover, the changes in soil quality were size-dependent, as canopy structure changed with cushion size. As a result of these biogenic environmental changes, the facilitative effect of *A. polytrichoides* cushions on the composition of the plant community and the biomass production of focal plants increased as the cushions increased in size. Our data, thus, provide evidence of a mechanistic link between ontogenetic development of cushion species and the biogenic environmental changes in abiotic factors, with relevant consequences for plant–plant interactions.

### Changes in Soil Properties with Cushion Size

In the HHM, the facilitative effect of *A. polytrichoides* cushions on other plant species was previously mainly attributed to fertility islands produced under their dense canopy (Yang et al., 2010; Chen et al., 2014). In addition, the distinct improvement in moisture and thermal conditions created by cushion species has been clearly reported for different mountain areas (Cavieres et al., 2006, 2008). However, data concerning the temporal dynamics of micro-environmental status within the compact structure of cushion species is very rare. On a spatial scale, the impact of *Arenaria tetraquetra* cushions on SOM and SWC have been reported to be greatest at high elevations where cushions were compact and large, while at low elevations where cushions were loose and small, these effects were much smaller (Schöb et al., 2013). Complementing these findings, here, at a single site, we observed that the effects of *A. polytrichoides* on soil properties changed with cushion size, with considerable increases in the main nutrients under large and compact cushions. All these findings together indicated that these components of microhabitat amelioration by cushions are highly likely to be dependent on their morphology (Schöb et al., 2013).

In semi-arid environments, facilitation resulting from improved nutrition under large (old) legume (*R. sphaerocarpa*) and non-legume (*Juniperus communis* subsp. *nana*) species has been attributed to a higher decomposition rate due to greater microbial activity and/or particular chemical or physical characteristics of litter under their canopies (Pugnaire et al., 1996; Allegrezza et al., 2016). Cushion species at high elevations have evolved their remarkable compact structure to trap and accumulate leaf litter and consequently create a constrained nutrition cycle via litter input (Körner, 2003). This suggestion has been supported by one recent study in the alpine zone of the Tibetan Plateau, where it was found that mineralization of cushion plant (*Androsace tapete*) litter during the winter may be an important source of N for plant growth early in the growing season and probably explains subsequent facilitation of cushion species in cold environments (He et al., 2014). Since litter fall is the source of the rich humus contained within the core of cushion plants and this comprises dead leaves and closely branching shoots with short internodes, litter accumulation should increase as the cushions develop. Meanwhile, the increased litter depth in large cushions observed in this study may suggest that more compact and larger cushions are likely to provide better shelter from the wind than loose and small cushions, thereby retaining more leaf litter and maintaining higher moisture content. These denser litter layers combined with higher water content within large cushions represent a favorable micro-environment for decomposition of litter (Schinner, 1982). Thus, as Pugnaire et al. (1996) found for *R. sphaerocarpa* in a semi-arid environment, the greatly improved nutrition under large cushions studied here could be related to higher litter turnover, a process which probably results in higher SOM below large cushions (Berendse, 1994). On the other hand, it has been suggested that cushions may need a certain range of trait values to create improved microhabitat conditions and act as nurses (Schöb et al., 2013). The comparatively limited improvements to microhabitat conditions achieved by the smallest cushions in our study therefore could be attributed to the limited litter accumulation, which restricts any biogenic changes in microhabitat conditions and soil properties, and in turn influences facilitation intensity (see below).

### Changes in Cushions’ Functional Traits with Size

The accumulation of resources (e.g., soil nutrients and water) facilitates the establishment and growth of other species in association with the *Arenaria* cushions; these changes also improve growing conditions for *Arenaria* itself in this challenging environment. There was a positive correlation between LMA and canopy size of *Arenaria* cushions. Although LMA alone is not always the best predictor of plant performance, higher LMA reflects better fitness of large and compact cushions under adverse growing conditions (reviewed by Poorter et al., 2009). Additionally, a higher LDMC was also observed in large and compact cushions. Leaves with high LDMC tend to be relatively tough and are less likely to suffer physical damage (e.g., wind and hail, Pérez-Harguindeguy et al., 2003). However, not all examined parameters responded linearly with size in our study. Foliar δ13C was found to be unchanged in relation to the size-classes of *Arenaria* cushions, which may suggest that carbon isotope discrimination is uncoupled from ontogenetic development of this species. In desert environments, carbon isotope discrimination was found to be negatively correlated with plant size (Schuster et al., 1992). Given the wet monsoonal climate during the majority of the growing season within the HHM, *Arenaria* cushions must rarely suffer from water stress and thus their gas exchange physiology probably remains unchanged during their lifetime. Generally, fruit production has been shown to relate to plant size (Herrera, 1991; Zammir and Zedler, 1993; Greene and Johnson, 1994; Pugnaire et al., 1996). In this study, reproductive allocation, measured as the number of flowers per area, followed this pattern. The increased reproductive effort could be the result of greater availability of resources and a larger source (Pugnaire et al., 1996). It may imply a positive link between plant vigor and its ameliorative effects on microhabitats.

### Interaction between Cushions and Other Plant Species

The species richness/abundance and biomass of beneficiary species were found to increase with increasing cushion size. These intra-specific changes in facilitation intensity could have been the result of size-dependent amelioration of physical and chemical properties (particularly soil nutrients) in the upper soil layer by cushions. A similar size–age related interaction has also been reported in a semi-arid environment, where plant diversity in the understory of *R. sphaerocarpa* and *Juniperus communis* was found to increase with shrub size/age, probably due to greater heterogeneity under the larger canopy (Pugnaire et al., 1996; Allegrezza et al., 2016). In addition, one previous study of cushions in the same region as ours attributed interspecific differences (*Arenaria polytrichoides* vs. *Potentilla articulata*) in intensity of facilitation to distinct capacities for improving soil nutrients of two phylogenetically distant species but which shared similar cushion morphology (Chen et al., 2014). It has been suggested that young/small plant individuals can hardly act as effective nurse plants due to their restricted size, which limits their impact on micro-environmental conditions (Callaway, 2007). For *Juniperus* shrubs, the effects of a small canopy were mainly negative, resulting in almost complete exclusion of co-existing species beneath the shrub. Such an inhibitive effect was ascribed to the dramatic light depletion under small shrubs (Allegrezza et al., 2016). In contrast, a positive interaction was observed in the smallest *Arenaria* cushions in our study, although the overall effect was much less pronounced compared to that of the large cushions. This may be a direct consequence of improved soil properties created by the compact structure of cushions. However, the limited improvement in soil quality, in particular in terms of N availability, may have reduced the facilitation intensity in small sized cushions. Unlike the study of shrubs in a semi-arid ecosystem that reported a transition of the nurse shrub (*S. spinosum*) from a ‘young’ to an ‘old’ stage that shifted the species from facilitation to interference because of the extreme shade under the canopy of oldest plants (Reisman-Berman, 2007), *A. polytrichoides* cushions in our study exhibit a monotonic facilitation pattern with respect to other plant species. Seedlings in the shrub patches of arid environments could experience dual stress resulting from limited light and water availability. It seems that in these arid environments, the benefits of improving water relations under the canopy exceeds the costs associated with growing at an extremely low light level (Holmgren et al., 1997). In contrast, the facilitation by *Arenaria* cushions at our study site was mainly related to nutrient enrichment (Yang et al., 2010). Establishment and growth of plant seedlings in alpine regions are controlled by nutrient availability (Chambers et al., 1990). As the difference between soil properties under and outside cushions increased with cushion size, the range of environmental variation also increased, allowing establishment of more plant individuals and production of more biomass in association with the largest cushions. However, facilitation by cushion species in alpine areas is likely due to several factors acting in concert. One recent study reported a strong positive effect of root fungal endophytes from *Laretia acaulis* cushions on the performance and fitness of beneficiary species in high Andes in Central Chile (Molina-Montenegro et al., 2015). These findings suggest an important role of microorganism associations in mediating facilitation of cushion species. In a semi-arid environment in south-east Spain, soil microbial community under a nurse leguminous shrub, *Retama sphaerocarpa*, changed in compositions, biomass and activity as the nurse grows (Hortal et al., 2013). Future studies should consider changes in structure and compositions of soil microbial community with increasing size of the nurse cushion species and their influences on interaction between cushion species and other non-cushion species.

## Conclusion

The contribution of facilitation by cushion species to natural plant community properties is commonly discussed in the context of its importance along spatial stress gradients (e.g., Cavieres and Badano, 2009; Cavieres et al., 2014; Chen et al., 2015). Here at a fine spatial scale, however, we show that facilitation depends on the development of the cushion species acting as nurse plants as they grow in size. The size-dependent facilitation patterns observed here advance our understanding of biotic interactions on the temporal scale. One recent study indicated that the presence of mature nurse cushion plants in the sub-Antarctic region improves the demographic performance of an intermediately sized beneficiary but that these facilitation effect wane as the beneficiary develops further (le Roux et al., 2013). Our study demonstrates how size-dependent changes in nurse cushions during their development alter their favorability as sites for the establishment and growth of other plant species. Taken together, these findings demonstrate that size-dependent changes during benefactor or beneficiary plant ontogeny may shift the nature or intensity of interactions and thereby determine population dynamics over time.

## Author Contributions

YY and J-GC conceived and designed the experiments. YY and J-GC performed experiments and analyzed the data. YY wrote the manuscript. CS and HS provided editorial advice on experiments and writing of the manuscript.

## Conflict of Interest Statement

The authors declare that the research was conducted in the absence of any commercial or financial relationships that could be construed as a potential conflict of interest.
