# Supplementary material for: Size-Mediated Interaction between a Cushion Species and Other Non-cushion Species at High Elevations of the Hengduan Mountains, SW China
Source: Front Plant Sci. 2017 Apr 5;8:465. doi: 10.3389/fpls.2017.00465 (PMC5380752; doi:10.3389/fpls.2017.00465)
Supplement: Supplementary file 1 [file Table_1.DOCX]

***Supplementary Material***

**Size-mediated interaction between cushion species and other plant species at high elevations of the Hengduan Mountains, SW China**

Yang Yang^1*^, Jian-Guo Chen^1^, Christian Schöb^2^, Hang Sun^1*^

^1^ Key Laboratory for Plant Diversity and Biogeography of East Asia, Kunming Institute of Botany, Chinese Academy of Sciences, Kunming 650201, Yunnan, People’s Republic of China

^2^Department of Evolutionary Biology and Environmental Studies, University of Zürich, 8057 Zürich, Switzerland

*Correspondence:

Dr. Yang Yang

Key Laboratory for Plant Diversity and Biogeography of East Asia, Kunming Institute of Botany, Chinese Academy of Sciences, Kunming 650201, Yunnan, People’s Republic of China

[yangyang@mail.kib.ac.cn](mailto:yangyang@mail.kib.ac.cn);

Prof. Dr. Hang Sun

Key Laboratory for Plant Diversity and Biogeography of East Asia, Kunming Institute of Botany, Chinese Academy of Sciences, Kunming 650201, Yunnan, People’s Republic of China

[sunhang@mail.kib.ac.cn](mailto:sunhang@mail.kib.ac.cn)

**Supplementary Table 1**. Non-cushion species and their difference in abundance and biomass production quantified by RII­_abundance_ and RII_biomass_ between sampled patches of cushions and paired open areas in four different size classes.

|  | RII_abundance_ | | | | RII_biomass_ | | | |
| --- | --- | --- | --- | --- | --- | --- | --- | --- |
| Species | Class I  (10 cm) | Class II  (15 cm) | Class III  (20 cm) | Class IV  (30cm) | Class I  (10 cm) | Class II  (15 cm) | Class III  (20 cm) | Class IV  (30cm) |
| *Anaphalis viridis* | 0.20 | 0.50 | 0.14 | -1.00 | 0.26 | 0.98 | -0.35 | -1.00 |
| *Androsace zambalensis* | -0.50 | 0.56 | -1.00 | 0.43 | -0.23 | 0.17 | -1.00 | -0.19 |
| *Arenaria barbata* | -1.00 | -1.00 | -1.00 | -1.00 | -1.00 | -1.00 | -1.00 | -1.00 |
| *Aster tsarugensis* | NA | 1.00 | 0.52 | 0.60 | NA | 1.00 | 0.47 | 0.15 |
| *Chionocharis hookeri* | 0.00 | NA | NA | NA | -0.86 | NA | NA | NA |
| *Chesneya nubigena* | 0.60 | -0.50 | 0.85 | 0.81 | 0.68 | -0.46 | 0.90 | 0.84 |
| *Cyananthus macrocalyx* | 0.33 | NA | 1.00 | 1.00 | 0.72 | NA | 1.00 | 1.00 |
| *Gentiana caelestis* | NA | NA | 1.00 | -0.20 | NA | NA | 1.00 | 0.09 |
| *Kobresia cuneata* | 0.64 | 1.00 | 0.93 | 1.00 | 0.55 | 1.00 | 0.94 | 1.00 |
| *Koenigia islandica* | NA | -1.00 | -1.00 | -1.00 | NA | -1.00 | -1.00 | -1.00 |
| *Juncus przewalskii* | -1.00 | NA | NA | NA | -1.00 | NA | NA | NA |
| *Lagotis alutacea* | 0.00 | 0.00 | -0.47 | -0.07 | 0.26 | -0.18 | -0.26 | 0 |
| *Nardostachys jatamansi* | NA | NA | NA | 1.00 | NA | NA | NA | 1.00 |
| *Pedicularis likiangensis* | NA | NA | NA | -0.20 | NA | NA | NA | -0.47 |
| *Poa alpina* | NA | 0.00 | -1.00 | NA | NA | 0.33 | -1.00 | NA |
| *Polygonum macrophyllum* | 0.21 | 0.53 | 0.84 | 0.96 | 0.28 | 0.53 | 0.82 | 0.95 |
| *Potentilla coriandrifolia* | 0.23 | 0.69 | 0.78 | 0.94 | 0.01 | 0.70 | 0.88 | 0.96 |
| *Pyrethrum tatsienense* | 0.20 | 0.38 | 0.41 | 0.71 | 0.42 | -0.14 | 0.70 | 0.82 |
| *Rhdodendron impeditum* | NA | NA | NA | 1.00 | NA | NA | NA | 1.00 |
| *Rhodiola coccinea* | NA | 0.17 | 0.20 | -0.13 | NA | -0.02 | 0.61 | 0.57 |
| *Saussurea loriformis* | 0.20 | 1.00 | 0.90 | 1.00 | -0.28 | 1.00 | 0.96 | 1.00 |
| *Saussurea ochrochlaena* | NA | 1.00 | NA | 1.00 | NA | 1.00 | NA | 1.00 |
| *Saussurea wernerioides* | NA | NA | NA | 1.00 | NA | NA | NA | 1.00 |
| *Saxifraga aristulata* | -1.00 | -1.00 | 0.64 | -1.00 | -1.00 | -1.00 | 0.47 | -1.00 |
| *Sedum oreades* | 1.00 | -1.00 | -0.50 | 0.11 | 1.00 | -1.00 | 0.79 | 0.61 |
| *Sibbaldia purpurea* | 0.85 | 1.00 | 0.50 | 0.72 | 0.70 | 1.00 | 0.28 | 0.74 |
| *Solms-laubachia eurycarpa* | -0.20 | NA | 1.00 | 1.00 | 0.03 | NA | 1.00 | 1.00 |
| *Viola psedo-bambusetorum* | NA | NA | 0 | 0.85 | NA | NA | 0.20 | 0.73 |
| Percentage (%) of species exhibited positive values and total species recorded in cushions and paired open areas in each of the four size classes | 58.8%=(10/17)×100%= | 61.1%=(11/18) ×100% | 66.7% = (14/21) ×100% | 68.0%= (17/25) ×100% | 64.7%=(11/17) ×100% | 55.6% =(10/18) ×100% | 71.4% =(15/21) ×100% | 72.0%= (18/25) ×100% |
